# Molecular regulation of fruit ripening

**DOI:** 10.3389/fpls.2013.00198

**Published:** 2013-06-14

**Authors:** Sonia Osorio, Federico Scossa, Alisdair R. Fernie

**Affiliations:** ^1^Max-Planck-Institut für Molekulare PflanzenphysiologiePotsdam-Golm, Germany; ^2^Departamento de Biología Molecular y Bioquímica, Instituto de Hortofruticultura Subtropical y Mediterránea “La Mayora”, Consejo Superior de Investigaciones Científicas, Universidad de MálagaMálaga, Spain; ^3^Consiglio per la ricerca e la sperimentazione in agricoltura, Centro di ricerca per l’OrticolturaPontecagnano (Salerno), Italy

**Keywords:** climacteric fruit, non-climacteric fruit, hormones, ripening, regulation

## Abstract

Fruit ripening is a highly coordinated developmental process that coincides with seed maturation. The ripening process is regulated by thousands of genes that control progressive softening and/or lignification of pericarp layers, accumulation of sugars, acids, pigments, and release of volatiles. Key to crop improvement is a deeper understanding of the processes underlying fruit ripening. In tomato, mutations blocking the transition to ripe fruits have provided insights into the role of ethylene and its associated molecular networks involved in the control of ripening. However, the role of other plant hormones is still poorly understood. In this review, we describe how plant hormones, transcription factors, and epigenetic changes are intimately related to provide a tight control of the ripening process. Recent findings from comparative genomics and system biology approaches are discussed.

Fruits are a distinctive characteristic of Angiosperms. They occur today in a wide variety of forms and types. The ancestral fruit, dry and dehiscent, probably emerged in the early Cretaceous period; fleshy fruits appeared later in the Cretaceous or early Tertiary (Eriksson et al., 2000). The diversification of fruits from a dry dehiscent form to a fleshy drupe or berry, correlated with the rise of vertebrates, main agents of seed dispersal (Knapp, 2002). The maturation of fruits is a complex and highly coordinated developmental process. In fleshy fruits, ripening results in the production of succulent, flavorful, and soft pericarp that attract animals and facilitate seed dispersal (Giovannoni, 2001). In addition to softening, fruits normally exhibit increased accumulation of sugars, acids, pigments, and volatiles that increase interest and palatability to animals. Moreover, fruits are an important source of supplementary diet, providing minerals, vitamins, fibers, and antioxidants for humans. From an agronomical point of view, nutritional value, flavor, processing qualities, and shelf-life determine the quality of fruits.

The main changes associated with ripening include color (loss of green color and increase in non-photosynthetic pigments that vary depending on species and cultivar), firmness (softening by cell wall degrading activities and alterations in cuticle properties), taste (increase in sugar and decline in organic acids), and flavor (production of volatile compounds providing the characteristic aroma).

Analytical tools that allow comprehensive phenotyping at the level of transcriptome (Alba et al., 2005; Vriezen et al., 2008; Matas et al., 2011; Rohrmann et al., 2011), proteome (Lee et al., 2004; Rose et al., 2004; Saravanan and Rose, 2004), and metabolome (Fait et al., 2008; Lombardo et al., 2011) facilitate an overview of the metabolic network (Carrari et al., 2006; Deluc et al., 2007; Grimplet et al., 2007; Enfissi et al., 2010; Zamboni et al., 2010; Osorio et al., 2011, 2012; Rohrmann et al., 2011; Lee et al., 2012; Pan et al., 2013); whilst network analysis is beginning to yield a detailed understanding of the systems regulation underlying fruit development.

## HORMONAL AND TRANSCRIPTIONAL REGULATION DURING RIPENING

Fruits are generally classified into two physiological groups, climacteric and non-climacteric, according to their respiratory activity and associated ethylene biosynthesis profiles during ripening. Ethylene synthesis in climacteric fruits such as tomato, apple, and banana, is essential for normal fruit ripening and blocking either synthesis or perception of this hormone prevents ripening (Hamilton et al., 1990; Oeller et al., 1991; Rottmann et al., 1991; Barry et al., 1996).

Efforts to uncover the transcriptional regulation underlying carpel and fruit development were first focused on the dry dehiscent siliques of the model plant *Arabidopsis* (Liljegren et al., 2000; Dinneny et al., 2005). These studies clarified the role of several MADS-box transcription factors in tissue specification and mechanism of dehiscence. Among these, the redundant *SHATTERPROOF* 1/2 genes (*SHP*, members of the *AGAMOUS* subfamily) specified valve margin identity in the silique: when mutated, fruits became indehiscent. However, despite the striking anatomical differences between dry and fleshy fruits, subsequent studies, primarily focused on tomato, have shown the involvement in ripening regulation of several orthologs of those MADS-box genes previously characterized in *Arabidopsis* (Pnueli et al., 1994; Itkin et al., 2009; Vrebalov et al., 2009; Giménez et al., 2010; Bemer et al., 2012). It is now clear that a part of the regulatory networks underlying fruit development have been conserved during the evolution of fleshy fruits (Smaczniak et al., 2012; Seymour et al., 2013).

A number of important advances in our understanding of mechanisms that regulate ripening have also come from the characterization of monogenic tomato mutants, including ripening-inhibitor (*rin*), non-ripening (*nor*), colorless non-ripening *(Cnr*), green-ripe (*Gr*), green flesh (*gf*), high pigment 1 (*hp1*), high pigment 2 (*hp2*), and never-ripe (*Nr*; Lanahan et al., 1994; Mustilli et al., 1999; Vrebalov et al., 2002; Liu et al., 2004; Barry and Giovannoni, 2006; Manning et al., 2006; Barry et al., 2008). The *rin* mutant encodes a partially deleted MADS-box protein of the SEPALLATA clade (*SEP4*; Hileman et al., 2006), whereas *Cnr* is an epigenetic change which alters the promoter methylation of SQUAMOSA promoter binding (SPB) protein. NOR is a member of the NAC-domain transcription factor family (Giovannoni, 2007). A recent study in which the transcriptome, proteome, and targeted metabolite analysis were combined during development and ripening of *nor* and *rin* mutants, has helped to refine the ethylene-regulated expression of downstream genes and added to our knowledge the role of this hormone in both protein- and metabolite regulation in tomato ripening (Osorio et al., 2011). This data supported the view that *nor* and *rin* act together in a cascade to control ripening (Giovannoni et al., 1995; Thompson et al., 1999) and also suggested that *nor* has a more global effect on ethylene/ripening-related gene expression than *rin*, which indicates that *nor* likely operates upstream of *rin*. Recently, using a combined approach based on chromatin immunoprecipitation and transcriptome analysis, it was provided evidence that RIN interacts with the promoters of more than 200 genes, modulating the expression of its targets by activation or repression. RIN target genes are major regulators of ripening control, such as *CNR* and *NOR*, or belong (Martel et al., 2011) to well-known pathways active during the transition from green to ripe fruits (e.g., carotenoid accumulation, chlorophyll breakdown, ethylene synthesis and perception; Fujisawa et al., 2013).

Fruits such as strawberry, citrus, and grape have been classified as non-climacteric, based on the lack of the respiratory burst and on the low endogenous production of ethylene compared to standard climacteric fruits (Perkins-Veazie, 1995). In pepper fruits, some cultivars seem to be ethylene-insensitive, while others pepper cultivars treated with exogenous ethylene were able to stimulate the expression of ripening-specific genes (Armitage, 1989; Ferrarese et al., 1995; Harpster et al., 1997; El-Kereamy et al., 2003).

In strawberry, which has emerged as a prime model of non-climacteric fruit ripening, ethylene is relatively high in green fruits, decreases in white fruits, and finally increases again at the red stage of ripening (Perkins-Veazie et al., 1996; Iannetta et al., 2006). Interestingly, this last increase is accompanied by an enhanced respiration rate that resembles the one that occurs in climacteric fruits at the onset of ripening (Iannetta et al., 2006). For better understanding the function of ethylene during strawberry ripening, different approaches have been used. External application of ethylene caused the down-regulation of several cell wall-related genes, such as β-galactosidase, pectin methylesterase, or β-xylosidase (Trainotti et al., 2001; Castillejo et al., 2004; Bustamante et al., 2009), while the expression of other genes such as expansin, *FaEXP2* (Civello et al., 1999) was ethylene-insensitive. Recent studies at transcriptomic and metabolomic levels in transgenic strawberry fruits with decreased ethylene sensitivity indicates that ethylene action is required for normal fruit development, acting differently in the two parts of strawberry fruit, achenes and receptacle (Merchante et al., unpublished data). These results show that, although not as relevant as in climacteric fruits, ethylene may nevertheless play a role in strawberry fruit ripening.

**FIGURE 1:**
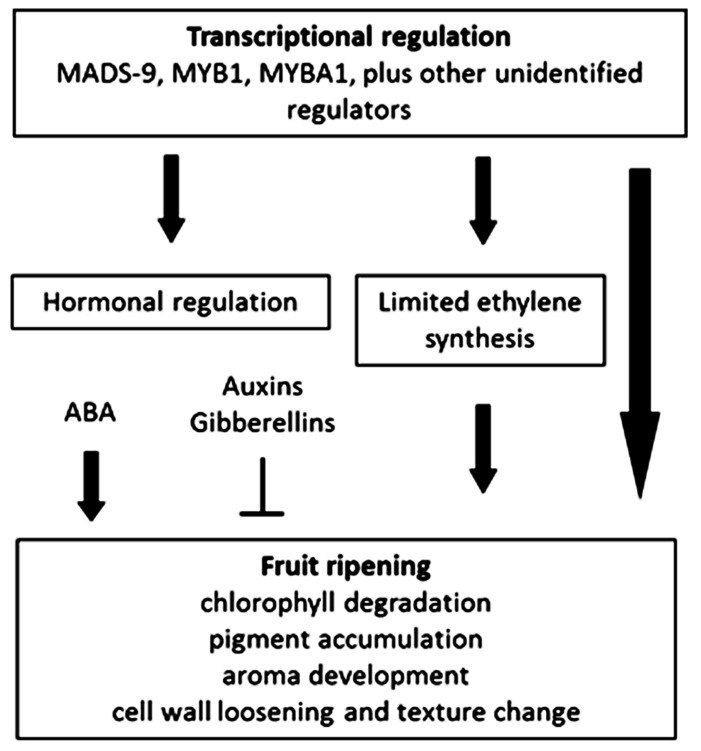
**Overview of ripening regulation in non-climacteric fruits**.

Recent comparative transcriptome and metabolome studies during the maturation processes of climacteric and non-climacteric fruits (tomato and pepper, respectively) suggest that both species have similar ethylene-mediated signaling components. In pepper, the regulation of these genes is, however, clearly different and may reflect altered ethylene sensitivity or regulators other than ethylene than in tomato (Osorio et al., 2012). Unlike the situation described in tomato the ethylene biosynthesis genes, aminocyclopropane-1-carboxylic acid (ACC) synthase, and ACC oxidase, are not induced in pepper. However, genes downstream of ethylene perception, such as cell wall-related genes, ethylene response factor 3 (*ERF3*), and carotenoid biosynthesis genes, are up-regulated during pepper fruit ripening (Osorio et al., 2012). Other commonly regulated genes between climacteric and non-climacteric fruits have been described. In strawberry, a *SEPALLATA* gene (*SEP1/2*; MADS-box) is needed for normal development and ripening (Seymour et al., 2011). Similarly, in banana, which is classified as a climacteric fruit, the MADS-box *SEP3* gene also displays ripening-related expression (Elitzur et al., 2010). In apple, *MADS2* gene expression is also associated with fruit firmness (Cevik et al., 2010), whereas in bilberry fruit, the *SQUAMOSA* MADS-box ortholog of the *TDR4* gene in tomato, has a role in regulation of anthocyanin biosynthesis (Jaakola et al., 2010; see **Figure 1**).

Current knowledge about the role of hormones – other than ethylene – in the development and ripening of climacteric and non-climacteric fruits is limited. In tomato, pepper, banana, muskmelon, and strawberry, the most abundant free auxin, indole-3-acetic acid (IAA), has been reported to decline prior to the onset of ripening; this reduction was accompanied by an increase of its conjugated form (IAA-Asp; Bottcher et al., 2010). The conjugation reaction is catalyzed by the IAA-amino synthase gene (*GH3*). In tomato, 15 members of *GH3* gene family have been described, but only for two of them is the pattern of expression associated with ripening (Kumar et al., 2012). Tomato fruits overexpressing the pepper *GH3* gene show anticipation of ripening (Liu et al., 2005), which is in agreement with the view that the ratio between IAA and its AA-conjugated form, rather than the level of IAA itself, may contribute to the temporal regulation of ripening (Bottcher et al., 2010). In non-climacteric fruits, no single growth regulator appears to play a positive role analogous to that played by ethylene, but it has been observed that auxin can negatively control the ripening of some non-climacteric fruits. In strawberry, it has been shown that the expression of many ripening-specific genes can be down-regulated by treatments with an exogenous auxin. Also in grape auxin seems to play a negative role in the regulation of ripening with synthetic auxin treatments delaying the expression of a number of ripening-related genes (Davies et al., 1997).

As a consequence of the prominent role of auxin in the development and ripening of some non-climacteric fruits, little attention has been paid to possible roles of other plant hormones, such as gibberellins (GAs). However, in strawberry, it has been reported that external application of GA_3_ to ripening fruits caused a significant delay in the development of the red color (Martinez et al., 1996) and modified the expression of genes involved in cell enlargement (de la Fuente et al., 2006) and cell wall disassembly (Bustamante et al., 2009).

In plants, the phytohormone abscisic acid (ABA) is known to be involved in various aspects of plant growth, development, and responses to environmental stresses (Leung and Giraudat, 1998; Finkelstein and Rock, 2002; Himmelbach et al., 2003; Hirayama and Shinozaki, 2007). ABA promotes sugar accumulation in fleshy fruits (Yamaki and Asakura, 1991; Kobashi et al., 1999; Richings et al., 2000; Pan et al., 2005) and plays a role in the regulation of climacteric and non-climacteric fruit ripening (Coombe, 1992; Davies et al., 1997; Giovannoni, 2001; Rodrigo et al., 2003; Zhang et al., 2009; Sun et al., 2012). In tomato, the suppression of the gene that catalyzes the first step in ABA biosynthesis (*NCED1*, 9-cis-epoxycarotenoid dioxygenase), results in the down-regulation of some ripening-related cell wall genes, such as polygalacturonase and pectinmethylesterase, as well as an increase in firmness and longer shelf-life (Sun et al., 2012). Similarly, reduction of *NCED* expression correlates with retardation of ripening in strawberry (Jia et al., 2011). ABA is considered a ripening-inducer in strawberry and grape fruits (Chai et al., 2011; Jia et al., 2011). The mechanisms of ABA signaling is not known, however, in grape, analysis of the GH3 promoter identified ABRE-like elements, which may indicate that the ABA/auxin content ratio is related to the initiation of ripening (Perkins-Veazie, 1995; Jiang and Joyce, 2003; Bottcher et al., 2010).

**FIGURE 2:**
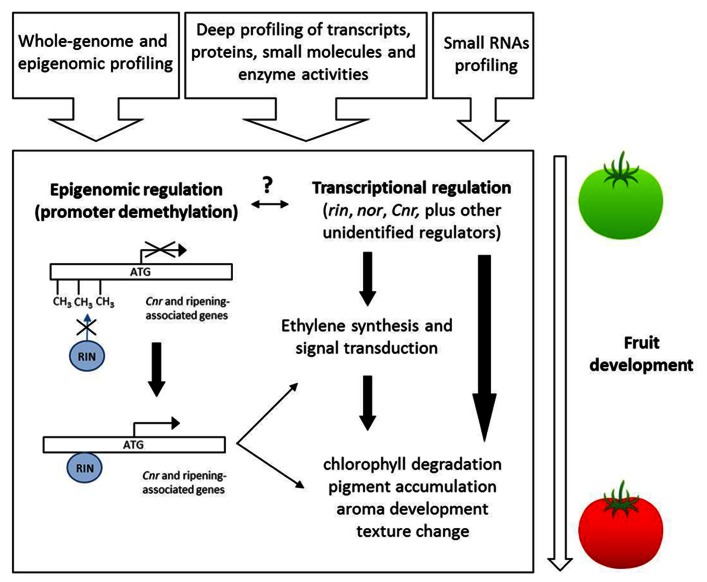
**Overview of ripening regulation in climacteric fruits.** The contribution of systems profiling approaches (shown at the top) will help identify novel regulatory genes and elucidate the interplay between epigenomic remodeling and transcriptional regulation involved during the ripening process.

In recent years, the level of understanding of the molecular events at the transcriptional, biochemical, hormonal, and metabolite levels underlying ripening in climacteric and non-climacteric fruits has increased considerable (see **Figures 1 and 2**). However, we still poorly understand the developmental switch that occurs in hormone responsiveness during the transition from immature to ripe fruits. To date, most published studies of transcriptional and metabolic regulation are of relatively low resolution at both spatial and temporal levels and are furthermore restricted in coverage of various cell molecular entities. However, new emerging technologies as well as improved statistical tools (Klie et al., 2011) allow us to further refine our analytical ability in order to cope with issues as subcellular compartmentation and contrasting behavior of different cell types (Caldana et al., 2012). Additionally, the availability of high quality fruit genome sequence data (Jaillon et al., 2007; Shulaev et al., 2011; Tomato Genome Consortium, 2012) will aid our understanding about the genetic regulation of fruit development and ripening.

## EPIGENETIC REMODELING DURING RIPENING

Epigenetic regulation of gene expression (inheritance without an alteration in the primary DNA sequence) is increasingly recognized as mechanism for modulating genome activity. Naturally occurring epigenetic changes at a single gene locus in plants can result in heritable morphological variation without alteration of the underlying DNA sequence (Patterson et al., 1993; Cubas et al., 1999; Manning et al., 2006). DNA methylation is one form of epigenetic regulation. It is involved in transcriptional regulation, stress responses and furthermore plays a major role in protecting the genome integrity against the activity of transposable elements (TEs) and other repetitive sequence (Chan et al., 2005).

In plants, DNA methylation occurs at cytosine residues in three different sequences (CG, CHG, and CHH, where H = A, C or T; Cokus et al., 2008) and is set in place and maintained by different factors (Law and Jacobsen, 2010).

Analysis of epigenetic variation in *Arabidopsis* revealed that at least one-third of expressed genes are methylated in their coding region, and only 5% of genes are methylated within promoter regions (Zhang et al., 2006; Vaughn et al., 2007). However, the promoter-methylated genes have a higher degree of tissue-specific expression (Zhang et al., 2006; Zilberman and Henikoff, 2007).

The first survey of the frequency and distribution of cytosine methylation sites in tomato dates back to more than 20 years ago, when it was found that polymorphisms in cytosine methylation between two tomato species were relatively abundant and that methylation patterns were stably inherited, from parents to offspring, segregating in a Mendelian fashion. The presence of tissue-specific methylation patterns and the overall decrease of 5-mC frequency in developing tissues also led the authors to postulate variation of methylation status of selected alleles during plant development (Messeguer et al., 1991).

More recently, the impact of cytosine methylation on tomato fruit ripening has strikingly emerged in the definition of the molecular nature of the colorless non-ripening phenotype. The tomato non-ripening *Cnr* mutant fails to produce ripe berries; fruits exhibit green pericarps and do not respond to external applications of ethylene. The gene at the *Cnr* locus was identified as a SPB protein-like using positional cloning, but the non-ripening phenotype could not be attributed to any alteration in the coding gene sequence. Bisulfite sequencing of the *Cnr* mutant allele showed instead hypermethylation of cytosine in the region upstream the predicted ATG start site. This hypermethylation state correlated with a drastic reduction of *Cnr* gene expression (Manning et al., 2006). Therefore, the non-ripening phenotype was due to the heritable cytosine hypermethylation pattern of the region including the *Cnr* gene promoter. Additionally, in normal tomato fruit (cv. Liberto) development, the promoter of *Cnr* appears to be demethylated in a specific region just prior to the onset of ripening. This lead to the hypothesis that DNA methylation contributes to the regulation of fruit ripening (Seymour et al., 2008).

Recent work by Zhong et al. (2013) provides genome-wide insights into the link between the genetic program of fruit ripening and DNA methylation state. On the basis of the previous results on the nature of the *Cnr* (epi) mutation, the authors injected a chemical inhibitor of cytosine methylation, 5-azacytidine, directly in the locular spaces and columella of developing tomato fruits. The methylation inhibitor induced the formation of local ripe areas, red in appearance, where the expression of typical ripening-related genes (phytoene synthase 1 and polygalacturonase) was anticipated. Moreover, the *Cnr* promoter region was demethylated in red sectors with respect to green parts of the fruits, pointing at the demethylation of *Cnr* as the epigenetic signal sufficient to induce ripening. The authors then extended their views on the role of cytosine methylation reporting the full tomato methylome sequences of leaves, immature and ripe fruits, including the ripening-impaired mutants *Cnr* and *rin*. The sequencing of the entire epigenome revealed at least three important results: (i) in wild-type fruits, the degree of methylation of regions upstream the transcription start sites (TSS) decreased gradually along fruit development; (ii) this general decline was not observed for the fruits of the ripening-impaired mutants *Cnr* and *rin*, whose CG methylation levels were constantly higher at TSS and, for *Cnr*, also comparable to those observed in leaves; (iii) the promoters of typical ripening-related genes were gradually demethylated during development of wild-type fruits. Further evidences about the link between ripening and cytosine methylation came from the ChIP-Seq mapping of RIN binding sites during fruit development. The set of RIN targets included 292 genes with a known role in ripening. RIN binding sites were found to be adjacent or overlapping the methylation “hotspots” upstream the TSS. The analysis of methylation status of these regions showed that they were progressively demethylated during the transition from green to red ripe fruits; and this lower level of methylation correlated with higher transcript levels of RIN target genes. A previous study showed that the binding of RIN to a limited set of promoters was inhibited in the *Cnr* epimutant, indicating that promoter hypermethylation may prevent RIN binding (Martel et al., 2011). These three main findings, i.e., that: (i) local treatment of immature fruits with a DNA demethylating chemical accelerates ripening; (ii) promoters of ripening genes, which contain RIN binding sites, are gradually demethylated during ripening but remain stably hypermethylated in ripening-deficient mutants; (iii) RIN does not bind hypermethylated *Cnr* gene promoters (and, possibly, all hypermethylated promoters of its target genes), taken together, assign a key role to the epigenome structure and developmental dynamics in coordinating tomato fruit ripening. The global scenario presented so far also suggests that progressive demethylation of ripening-related gene promoters may be the necessary condition for binding of transcriptional regulators, thus triggering the accumulation of ripening-related transcripts. In normal fruit development, however, the mechanism inducing demethylation of promoters remains elusive, and further efforts are needed in this direction to uncover the “missing link.” Given the growing importance of epigenetic modifications in impacting fruit phenotypes, we envisage that, in the future, high-throughput sequencing technologies will allow routine screening of crop epigenomes, accelerating detection of epigenetic variation. We anticipate that screening epigenome structure and dynamics will coexist with the analysis of conventional genetic variation in future plant breeding strategies. Epigenetic-based crop improvement approaches may radically impact fruit quality traits, especially for those traits whose allelic variation has been reduced during domestication or recent intensive breeding pressure. As such future modeling work aimed at integrating epigenomic profiling and small RNA profiling alongside the more frequently used transcript, protein, enzyme, and metabolite profiling (as suggested in **Figure 2**) will allow far greater understanding of the complex dynamics underlying this tightly regulated biological process.

## Conflict of Interest Statement

The authors declare that the research was conducted in the absence of any commercial or financial relationships that could be construed as a potential conflict of interest.
